# Microparticles Loaded with *Bursera microphylla* A. Gray Fruit Extract with Anti-Inflammatory and Antimicrobial Activity

**DOI:** 10.3390/ph17121565

**Published:** 2024-11-21

**Authors:** Víctor Alonso Reyna-Urrutia, Ramón Enrique Robles-Zepeda, Miriam Estevez, Marlen Alexis Gonzalez-Reyna, Grecia Vianney Alonso-Martínez, Juan Ramón Cáñez-Orozco, Julio César López-Romero, Heriberto Torres-Moreno

**Affiliations:** 1 Department of Chemical Biological and Agricultural Sciences, University of Sonora, Avenida University and Irigoyen, Caborca 83600, Sonora, Mexico; alonso.reyna@unison.mx (V.A.R.-U.); a220219607@unison.mx (G.V.A.-M.); 2 Department of Chemical Biological Sciences, University of Sonora, Blvd. Luis Encinas y Rosales, Hermosillo 83000, Sonora, Mexico; robles.zepeda@unison.mx (R.E.R.-Z.); a210218361@unison.mx (J.R.C.-O.); 3 Center for Applied Physics and Advacend Technologya, National Autonomous University of Mexico, Juriquilla Campus, Juriquilla 76230, Queretaro, Mexico; miries@fata.unam.mx (M.E.); marlenreyna@gmail.com (M.A.G.-R.)

**Keywords:** *Bursera microphylla*, chitosan, microparticles, encapsulation and bioactivity

## Abstract

**Background**: *Bursera microphylla* (B) A. Gray, a plant native to northwest Mexico, has long been utilized in traditional medicine for its anti-inflammatory effects. Previous studies have highlighted the bioactivity of *B. microphylla* fruit extract. Chitosan (Cs), a biopolymer known for its favorable physicochemical properties, has proven effective in encapsulating bioactive compounds. This study aimed to synthesize and characterize Cs-based microparticles containing *B. microphylla* fruit extract and evaluate their in vitro anti-inflammatory activity. **Methods:** Cs-based three-dimensional hydrogels were synthesized using physical cross-linking with ammonium hydroxide, incorporating *B. microphylla* fruit extract. The hydrogels were freeze-dried and mechanically ground into microparticles. The physicochemical properties of the microencapsulates were analyzed through scanning electron microscopy (SEM), optical microscopy (OM), Fourier transform infrared spectroscopy (FTIR), thermogravimetric analysis (TGA), and moisture absorption tests. Anti-inflammatory activity was assessed by measuring nitric oxide (NO) reduction in LPS-activated RAW 264.7 cells. Antimicrobial activity was evaluated against *Staphylococcus aureus*. **Results:** SEM and OM analyses revealed irregular morphologies with rounded protuberances, with particle sizes ranging from 135 to 180 µm. FTIR spectra indicated that no new chemical bonds were formed, preserving the integrity of the original compounds. TGA confirmed that the encapsulated extract was heat-protected. The moisture absorption test indicated the microparticles’ hydrophilic nature. In vitro, the microencapsulated extract reduced NO production by 46%, compared to 32% for the non-encapsulated extract. The microencapsulated extract was effective in reducing the microbial load of *S. aureus* between 15–24%. **Conclusions:** Cs-based microencapsulates containing *B. microphylla* fruit extract exhibited no chemical interactions during synthesis and demonstrated significant anti-inflammatory and antimicrobial activity. These results suggest that the Cs-based system is a promising candidate for managing inflammatory conditions.

## 1. Introduction

The multiplication of inflammation-related diseases has considerably increased the rate of prescription of non-steroidal anti-inflammatory drugs (NSAIDs) and corticosteroids [1,2]. Due to their effectiveness in reducing pain and inflammation, NSAIDs and corticosteroids are among the most used treatments for these conditions. However, the prolonged and excessive use of these drugs is related to a wide range of side effects, with gastrointestinal and cardiovascular complications being the most common [3,4]. For this reason, it is necessary to develop more effective anti-inflammatory agents with a better safety profile [5,6].

On the other hand, bacterial infections have become a severe public health challenge worldwide [7]. In this way, it is estimated that bacterial infections generated by antibiotic-resistant strains will constitute the greatest cause of death in the world in 2050 [8]. The resistance phenomenon makes traditional antibiotic treatments ineffective, causing infectious processes that are more difficult to treat. *S. aureus* is one of the most important microorganisms in the clinical area because it is one of the most common pathogens isolated in infectious processes [9]. In turn, it is important to mention that the World Health Organization (WHO) considers this pathogen a high-priority microorganism due to its high resistance characteristics to antibiotics [10]. For this reason, it is necessary to find new pharmacological strategies that can effectively treat bacterial infections.

In this sense, pharmaceutical engineering has opted to develop encapsulating systems from synthetic or natural polymers such as chitosan (Cs). This biomaterial is a polysaccharide industrially obtained from the alkaline deacetylation of crustacean chitin (exoskeleton of shrimp and crab) [11]. Cs is a non-toxic biopolymer with many biomedical applications (hemostatic, antimicrobial, cytocompatibility, biocompatibility, etc.). The Food and Drug Administration (FDA) considers this biopolymer as GRAS (Generally Recognized as Safe) with commercial availability at a low cost [12,13]. The cationic nature of Cs in acidic conditions favors the development of materials with different geometries, such as nano/microparticles, emulsions, fibers, hydrogels, films, and membranes. Cs, in its various forms, has been widely used as a matrix for the encapsulation of extracts, essential oils, and bioactive compounds [13,14,15]. Ionic gelation is one of the most promising encapsulation techniques since no chemical agents are used as cross-linking agents, and the biocompatibility of the polymeric matrix is not affected [15,16]. By enhancing the availability of the amino group in the Cs molecule, which is responsible for its bioactive properties and serves as the functional group used during chemical cross-linking, it becomes possible to obtain materials with unexplored stability and bioactivity [17]. In this way, by using ammonium hydroxide, physical bonds are generated that prevent cross-linking with the functional group, leaving the amino group free to be utilized for its bioactive properties. The materials are then lyophilized for preservation. For this reason, chitosan can serve as an encapsulating vehicle for the active compounds of medicinal plants.

*Bursera microphylla* A. Gray (*Burseraceae*) commonly named “torote”, “white torote”, “copal” or “elephant tree”, is a medicinal plant native to the desert region of northern Mexico and the southwestern United States, which covers the vast majority of the area geography of the Sonora Desert [18,19]. In traditional Mexican medicine, the Seri ethnic group makes use of the bark, fruits, and leaves of the plant to treat various disorders such as wound healing, sore throat, and headache. Currently, the preparation of tinctures based on *B. microphylla* has been reported as a remedy to control gum sores and teeth with abscesses. Stems and leaves of *B. microphylla* have also been used for the treatment of pain when urinating and as an anti-inflammatory [19,20].

In vitro studies demonstrate that *B. microphylla* fruit extracts inhibit the production of inflammatory mediators such as NO and TNF-α in RAW 264.7 cells activated with bacterial LPS [21,22]. However, no microsystem has been developed to encapsulate *B. microphylla* extract for therapeutic purposes. Due to the bioactive properties of *B. microphylla* extracts, the objective of this research was to synthesize and characterize a Cs-encapsulating polymeric system that maintains or enhances the properties of the extract. The function of these Cs microsystems is to potentiate the bioactivity of the extract by controlling its release, improving its bioavailability, and the absorption of bioactive compounds through the epithelial layers. This is reflected in the use of a smaller number of doses during therapy, which impacts the reduction in treatment costs. Furthermore, these microsystems reduce problems of physical or chemical instability of bioactive compounds by protecting them from unfavorable environmental conditions, reducing their volatilization and/or degradation [23]. The potential of these microparticles could be particularly relevant in treating chronic inflammatory conditions such as inflammatory bowel disease, which are areas of significant interest in pharmaceutical research.

## 2. Results and Discussion

### 2.1. Physicochemical Characterization of Microparticles

#### 2.1.1. Microparticle Morphology

**Figure 1:**
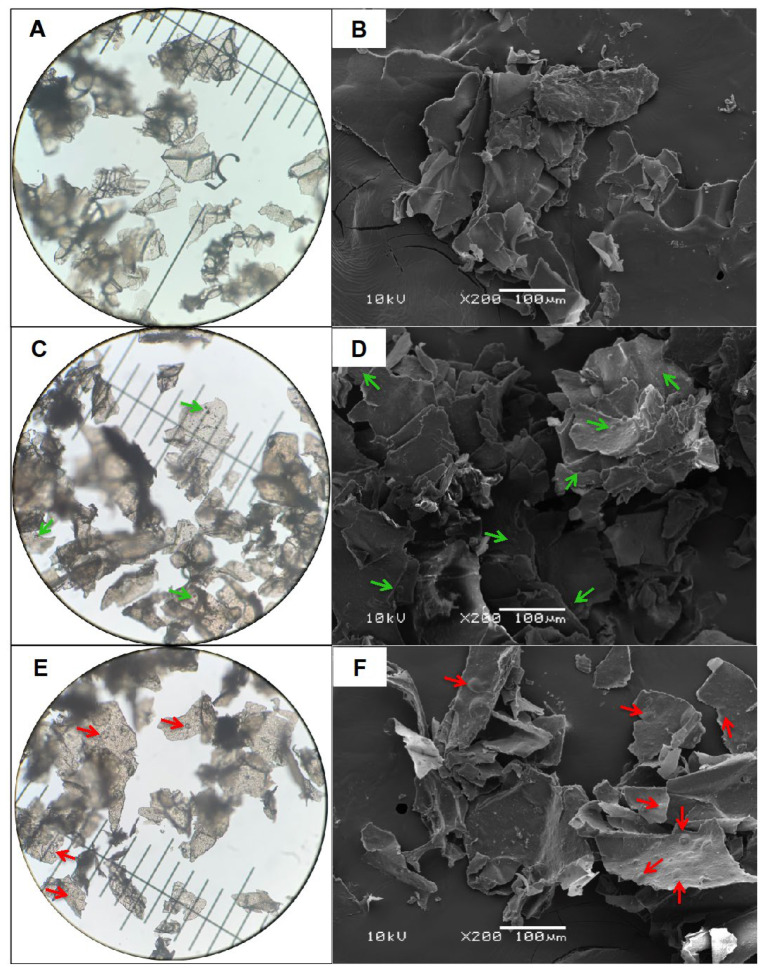
SEM micrographs (**right** column) at 200× and OM micrographs (**left** column) of microparticles. (**A**,**B**) Cs, (**C**,**D**) CsB-0.5 and (**E**,**F**) CsB-1.0. Cs: chitosan, CsB-0.5: chitosan-0.5% *Bursera microphylla* extract and CsB-1.0: chitosan-0.5% *Bursera microphylla* extract. Green and red arrows indicate *B. microphylla* extract present in the microparticles. (**A**,**C**,**E**; **left** column): 10 µm scale on the ruler.

The morphological characteristics of the microparticles (Cs, CsB-0.5 and CsB-1.0) are shown in Figure 1. By OM and SEM, it was observed that these materials presented a heterogeneous morphology with irregularities.

In CsB-0.5 (Figure 1C,D) marked with green arrows, round bodies can be observed in the walls that make up the three-dimensional network of the encapsulating microsystem. These findings were more evident in CsB-1.0 (Figure 1E,F), where larger rounded bodies were observed. In the microparticles loaded with 0.5% extract, it was possible to observe larger round bodies within the polymeric matrix; these changes were more evident in the 1.0% formulation (see red arrows). These findings suggest the presence of the extract in the walls that make up the three-dimensional network of the encapsulating microsystem (see red arrows). These findings indicate that the formation of round bodies could be associated with the incorporation of the extract into the encapsulating matrix.

#### 2.1.2. Microparticle Size Distribution and Specific Surface Area

**Figure 2:**
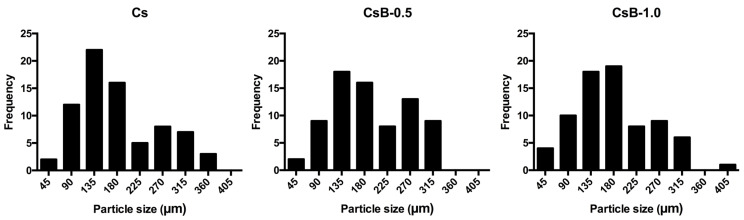
Particle size distribution of the microencapsulation of Cs, CsB-0.5 and CsB-1.0 of *B. microphylla*. Cs: chitosan microparticles; B: *B. microphylla* extract; CsB-0.5: chitosan microparticles loaded with 0.5% of *B. microphylla* extract; and CsB-1.0: chitosan microparticles loaded with 1.0% of *B. microphylla* extract.

Analysis of the size of the Cs microparticles showed a bimodal distribution, where the most abundant particle size frequencies were 135 µm and 270 µm (Figure 2). In contrast, microparticles loaded with *B. microphylla* fruit extract showed a single particle size distribution. CsB-0.5 microparticles presented a skewed distribution to the right with a predominant particle size frequency of 135 µm and 180 µm. Meanwhile, CsB-1.0 shows a slightly unimodal distribution skewed to the right, with a higher frequency of 180 microns. Although the microparticles presented non-uniform distributions, in all cases the most predominant distribution frequencies exceeded 50%.

The specific surface area (SSA) of the encapsulating systems is shown in Table 1. The SSA value was 110.5% higher for CsB-0.5 concerning Cs. Similarly, the SSA of CsB-0.5 was 45% higher compared to CsB-1. The higher SSA of CsB-0.5 could be related to the formation of small protuberances on the walls of the microparticles. On the other hand, the increase in the concentration of extract in CsB-1 produces larger protuberances, which causes the SSA to reduce. These treatments show behaviors with promising values that will benefit the bioactivity, contact area, and bioavailability of the extract incorporated into the designed microparticles. However, the SSA values of the microparticles with the *B. microphylla* extract are higher than those reported for other microencapsulations in which the major component is Cs as an encapsulating matrix or transport vehicle of a principle/drug. These values could indicate the bioavailability of our encapsulating systems, which acquire a high free contact area that may be required when coming into contact with physiological fluids, and the extract found in the three-dimensional structure of the particulate materials can be released to present adequate bioactivity [24,25,26,27]. Moreover, enhancing the bioavailability through controlled release mechanisms is crucial in developing more efficient drug delivery systems, potentially reducing dosage frequency and improving patient ease of treatment.

#### 2.1.3. Structure for the Fourier Transform Infrared Spectroscopy (FTIR)

**Figure 3:**
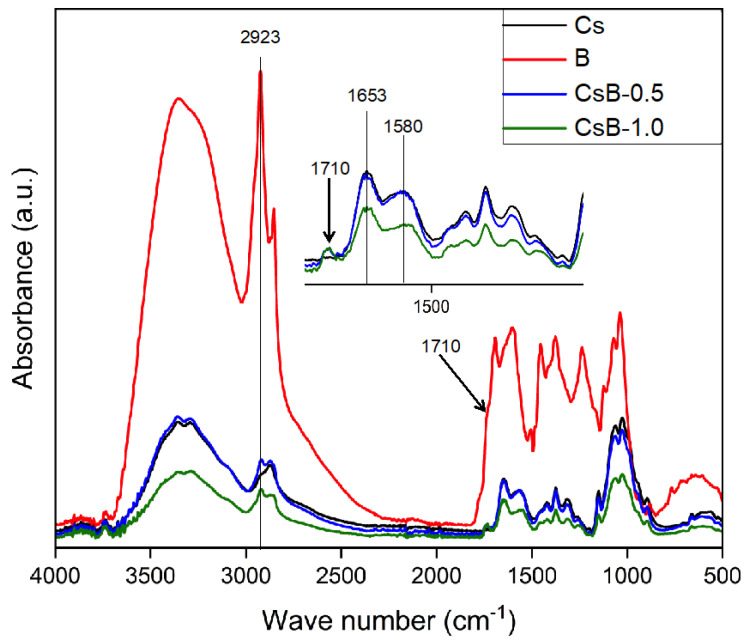
FTIR spectra of microencapsulation. Cs: chitosan microparticles; B: *B. microphylla* extract; CsB-0.5: chitosan microparticles loaded with 0.5% of *B. microphylla* extract; and CsB-1.0: chitosan microparticles loaded with 1.0% of *B. microphylla* extract.

Figure 3 presents the FTIR spectra of the microencapsulation. All spectra were similar, showing the characteristic absorption bands of Cs. In the wavenumber range of 3500 to 3300 cm^−1^, a relatively broad and intense band was observed, corresponding to the stretching vibrations of the O-H and N-H bonds. Peaks were also detected at 2923 and 2880 cm^−1^, associated with methylene groups, band at 1653 cm^−1^, which is associated with the C=O stretch of amide I and, at 1580 cm^−1^, related to the deformation of amide II [35,36,37] and, finally, in the range of 1200 to 500 cm^−1^, characteristic signals of the saccharide structure [38].

The microparticles loaded with *B. microphylla* extract (CsB-0.5 and CsB-1.0) showed a signal at 1710 cm^−1^ corresponding to the stretching vibration of C=O, characteristic of the aldehydes and ketones groups. Another signal with greater intensity that could be associated with the presence of the extract components in the microparticles was observed at 2923 cm^−1^ [39]. Comparative analysis of the rest of the FTIR signals shows that the extract-loaded microparticles and the unloaded microparticles present similar signal patterns, indicating that there is no presence of new functional groups in the loaded materials. The above confirms the absence of chemical interactions between Cs and the *B. microphylla* extract, suggesting that the components of the extract do not change in their chemical structure during the microparticle synthesis process, so the activity of the extract is not affected. When there is an interaction between the extract and chitosan, they make use of the free amino group of the chitosan polymer chains and this will stimulate a slow release caused by a degradation mechanism. On the other hand, if there is no interaction with the extract in which the encapsulation mechanism is entrapped in the chitosan polymer chains, a pH-triggered release is encouraged, dependent on the protonation/deprotonation of the chitosan amine groups after diffusion of the extract from the microparticle matrix [40].

#### 2.1.4. Thermogravimetric Analysis (TGA)

**Figure 4:**
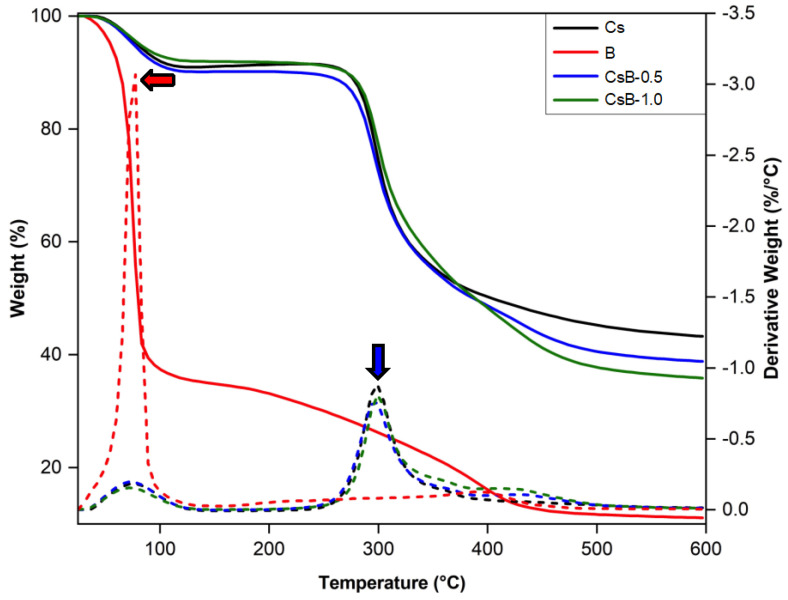
Thermogravimetric analysis of the synthesized microparticles. Cs: chitosan microparticles; B: *B. microphylla* extract; CsB-0.5: chitosan microparticles loaded with 0.5% of *B. microphylla* extract; and CsB-1.0: chitosan microparticles loaded with 1.0% of *B. microphylla* extract. Red arrow: Maximum loss of *B. microphylla* extract; Blue arrow: Maximum loss of Cs end Cs with *B. microphylla* extract. Dashed lines represent the derivative of the mass loss of the treatments.

The thermogravimetric analysis (Figure 4) compares the thermal stability of the microparticles with *B. microphylla* fruit extract. The extract showed a mass loss of ≈60% between 50–100 °C, with a maximum peak observed at 78 °C (see red arrow). On the other hand, for microparticles (Cs, CsB-0.5, and CsB-1.0), a mass loss of ≈6% was observed at ≈100 °C due to the loss of water molecules. The second mass loss for the microparticles occurred at 303 °C (see blue arrow), as a result of the degradation of the saccharides of the Cs molecule [41,42,43]. Similar behavior was observed in the microparticle loaded with *B. microphylla* extract (CsB-0.5% and CsB-1.0%), where the Cs matrix conferred thermal stability to the extract encapsulated in the walls of the polymeric matrix, helping to protect the extract from decomposition or degradation at high temperatures.

#### 2.1.5. Moisture Absorption

**Figure 5:**
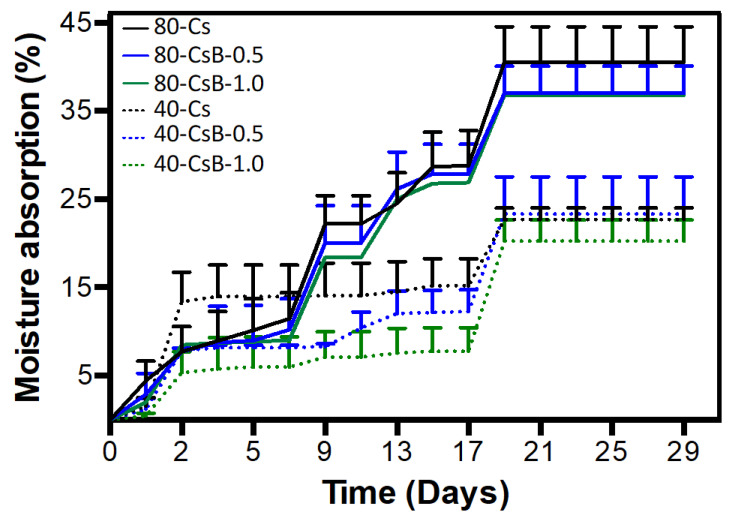
Moisture absorption of microparticles at a relative humidity of 40 ± 1% and 80 ± 1%. Values of *n* = 3 ± SD are shown. Cs: chitosan microparticles; B: *B. microphylla* extract; CsB-0.5: chitosan microparticles loaded with 0.5% of *B. microphylla* extract; and CsB-1.0: chitosan microparticles loaded with 1.0% of *B. microphylla* extract.

Moisture absorption analysis showed microparticles (Cs, CsB-0.5, and CsB-1.0) presented maximum moisture absorption of 20–22% at 40 ± 1% R.H. On the other hand, the maximum moisture absorption for all microparticles was found between 37–40% at 80 ± 1% R.H. (Figure 5). The behaviors were with values similar to those reported in the literature where Cs is the majority polymeric matrix of the material, achieving an equilibrium moisture absorption of 41% at high R.H. values [44]. These results demonstrate that microparticles have a hydrophilic character when exposed to high relative humidity. This property of the materials can be beneficial in terms of exposure to physiological fluids since when in contact with these systems, it causes a release of the extracts encapsulated in the polymeric matrix. It should also be mentioned that the hydrophilic character of the Cs matrix is conferred to the free amino group of the Chitosan polymeric chain, confirming the null chemical interaction between *B. microphylla* extracts and the Cs structural chain [45].

### 2.2. Bioactive Characterization of Microparticles

#### 2.2.1. Cytotoxic Effect

**Figure 6:**
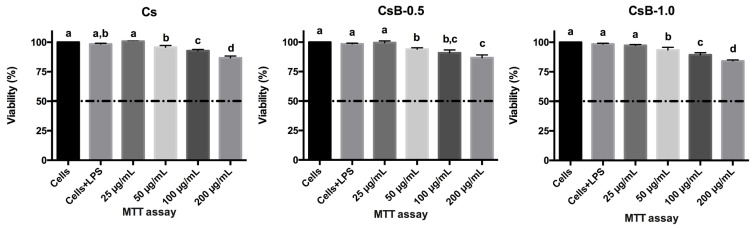
Cytotoxic effect of microparticles on RAW 264.7. Bars with different letters a–d indicate statistically significant differences (*p* < 0.05). All values represent the mean ± standard deviation of three independent experiments (±SD, *n* = 3). LPS = Lipopolysaccharide. Cs: chitosan microparticles; B: *B. microphylla* extract; CsB-0.5: chitosan microparticles loaded with 0.5% of *B. microphylla* extract; and CsB-1.0: chitosan microparticles loaded with 1.0% of *B. microphylla* extract.

Assessing the anti-inflammatory effect in cell models must be accompanied by determining the cytotoxic effect of the treatments, to guarantee that the effect is associated with the inhibition of the inflammation and not by the induction of cell death [46]. The results (Figure 6) showed that in general, microparticles did not show cytotoxic effects on RAW 264.7 cells. Except at (200 μg/mL) Cs, CsB-0.5 and CsB-1 reduced the cell viability by 87, 86 and 84%, respectively. These results are consistent with those reported by Torres-Moreno et al. (2022) for extracts of stems, fruits, and leaves of *B. microphylla,* where viability was at 90% in the different extract concentrations assessed in said research [21]. While the encapsulation of *B. microphylla* extract within chitosan microparticles shows promise, potential side effects such as immunogenicity or allergic reactions must be thoroughly evaluated in future studies to ensure patient safety. It should be mentioned that the cytotoxic effect of the investigation with the pure extract showed higher cytotoxic activity compared to the microencapsulation of the extract in the Cs matrix.

#### 2.2.2. Effect on the Nitric Oxide (NO) Production

**Figure 7:**
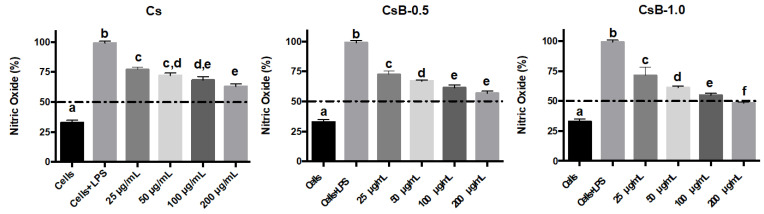
Effect of encapsulating systems of *B. microphylla* extracts of fruits on the production of NO in RAW 264.7-activated with LPS. Bars with different letters a–f indicate statistical differences (*p* < 0.05). All values represent the mean ± standard deviation of three independent experiments (±SD, *n* = 3). LPS = Lipopolysaccharide. Cs: chitosan microparticles; B: *B. microphylla* extract; CsB-0.5: chitosan microparticles loaded with 0.5% of *B. microphylla* extract; and CsB-1.0: chitosan microparticles loaded with 1.0% of *B. microphylla* extract.

Figure 7 shows the anti-inflammatory activity of the Cs encapsulating systems. The ability of these particulate materials to reduce NO production in RAW 264.7 macrophages activated with LPS was evaluated. The results show that stimulation of macrophages with LPS leads to a 3.2-fold increase in NO production compared to the basal level shown with untreated cells. In the cells treated with CsB-0.5 and CsB-1.0 (200 μg/mL), the NO levels were reduced by 39.4 and 46.0%, respectively. This effect was greater than that shown by the unloaded Cs microparticles, where at the maximum concentration tested (200 μg/mL), a reduction in NO production of 32% was observed. These effects are relatively lower compared to the observed for the extract because the microparticles contain 0.5% and 1% of the extract. Therefore, these findings suggest that the *B. microphylla* extract can be used more efficiently by incorporating it into the Cs matrix. [47]. This NO reduction effect is highly significant, as NO is a pro-inflammatory mediator involved in the development of various diseases of major concern to the healthcare sector, including diabetes, cardiovascular diseases, arthritis, allergies, and cancer, among others. This reduction could have broad functional applications in counteracting these conditions [48,49]. Similarly, it is relevant that it was possible to raise the IC_50_ of the *B. microphila* extract with values in its different seasonalities <200 µg/mL to values >200 µg/mL shown in the CsB-0.5% Ex encapsulant system [21]. The above is of great advantage since a smaller amount of encapsulated extract would be used in the microparticle to achieve the effectiveness of the extract. This increases the functionality of the extract combined with Cs to obtain microparticles loaded with the *B. microphylla* extract in a polymeric matrix with a synergistic behavior in its bioactivity.

#### 2.2.3. Antimicrobial Evaluation

**Figure 8:**
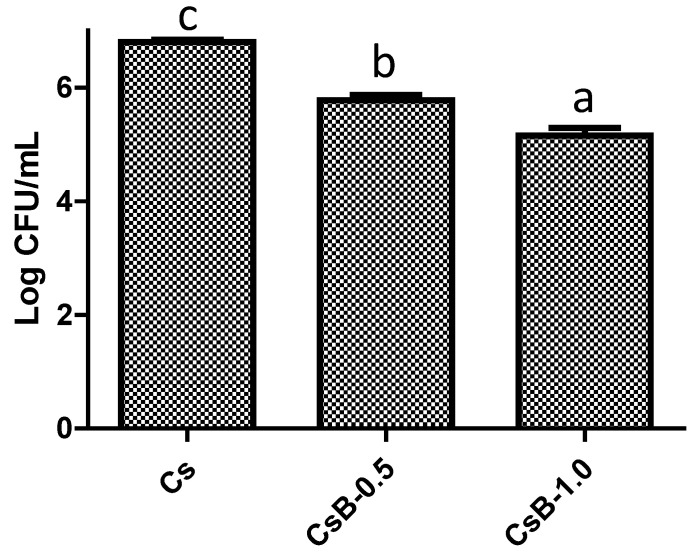
Antimicrobial activity of microparticles loaded with *B. microphylla* A. Gray fruit extract against *S. aureus*. Bars with different letters a–c indicate statistical differences (*p* < 0.05). All values represent the mean ± standard deviation of three independent experiments (±SD, *n* = 3). Cs: chitosan microparticles; B: *B. microphylla* extract; CsB-0.5: chitosan microparticles loaded with 0.5% of *B. microphylla* extract; and CsB-1.0: chitosan microparticles loaded with 1.0% of *B. microphylla* extract.

Results obtained in the antimicrobial evaluation of microparticles loaded with *B. microphylla* extract are presented in Figure 8. Results show that microparticles loaded with *B. microphylla* extract significantly reduce the microbial load in both treatments, with the CsB-1.0 treatment being the most effective, reducing 24% of the logarithmic count, followed by CsB-0.5 treatment, decreasing 15% of the logarithmic count. The results suggest that microparticles can release the extracts and their chemical compounds and interact with *S. aureus*, causing bacterial cell damage. The results presented in this study showed novel and original information because this is the first study that reports the antimicrobial activity of *B. microphylla*. Additionally, it is important to highlight that *S. aureus* is one of the most important Gram-positive bacteria in the health area because it is one of the main pathogens acquired and transmitted at clinical and community levels and also develops the ability to tolerate the effect of antimicrobial therapies [9]. The antimicrobial effect of *B. microphylla* can be associated with the presence of chemical compounds. Previous studies of our research group have demonstrated that *B. microphylla* fruit extract showed phenolic compounds such as flavonoids, phenolic acids, and lignans [21,22]. In this way, phenolic compounds have demonstrated a potent antimicrobial effect against *S. aureus*. The antimicrobial mode of action of phenolic compounds is related to their capacity to interact with bacterial enzymes and toxins, cell walls, and bacterial membranes, interfere with metabolic pathways, induce DNA fragmentation, and suppress virulence genes [50,51,52]. Based on the above, the results offered an important basis for future studies in which the concentration of *B. microphylla* extract in the microparticles can be increased to improve the antimicrobial effect. In addition, it seems feasible to carry out antimicrobial evaluations in other bacterial strains, such as antibiotic-resistant clinical isolates. The above is performed to use these polymeric matrices as possible alternatives for the development of pharmacological therapies.

## 3. Materials and Methods

### 3.1. Materials

Chitosan (Cs) with catalog No. 448877 was used to prepare the microparticles. Ethyl alcohol (EA, 94.9–96%, No. 493538) was used to obtain the plant extract of *Bursera microphylla*. Ammonium hydroxide (AH, 28% NH_3_ in H_2_O, ≥99.99% trace metals basis, No. 338818) and glacial acetic acid (AA, ≥99.5% purity No. 33209) were used to make the hydrogels. Magnesium nitrate hexahydrate (MNH, 99%, No. 237175) and sodium sulfate decahydrate (SSD, 99%, No. 403008) were used to measure relative humidity absorption. Griess reagent (catalog No. G4410), MTT (catalog No. 88417), and isopropanol (catalog No. 563935) were used in the in vitro assays. All solvents and reagents were purchased from Sigma-Aldrich (Toluca, Mexico).

### 3.2. Plant Material and Extract Elaboration

*Bursera microphylla* specimens were collected in the State of Sonora of Mexico in the “Puerto Lobos” area (30.177211, −112.664644) of the municipality of Caborca during the spring (30 April 2023). The collected fruits were weighed and placed in a flask with ethanol (1:10 *w/v*) at 25 °C for 10 days with daily manual shaking. The extracts were filtered and evaporated in an IKA rotary evaporator model RV-10B-S99 of 125 rpm, heating to 50 °C. Once the ethanolic extracts were obtained, they were dried and stored at 4 °C until use.

### 3.3. Obtaining Microparticles

Different solutions were prepared for the synthesis of microencapsulation with/without *Bursera microphylla* extract: (1) Cs solution at 2% (*w/w*) was prepared, dissolving in 0.3 M glacial acetic acid at room temperature then stirred until completely dissolved [53]. (2) Cs solutions were prepared with 0.5–1.0% *B. microphylla* extract, CsB-0.5 (99.5/0.5) and CsB-1.0 (99/01), ratio (*w/w*), respectively. Where the extract was mixed in the Cs solution for 30 min [54].

Aliquots of the polymer suspensions were made in 10 mL beakers and placed inside a hermetically closed chamber, containing 100 mL of ammonium hydroxide. Physical cross-linking (gelation) was induced by ammonia diffusion for 24 h [55]. Subsequently, the hydrogels were washed with distilled water to eliminate ammonium acetate (CH_3_COONH_4_), and the rest of the ammonium hydroxide (NH_4_OH) remained in the hydrogels until a pH of 7 was reached. Afterward, the hydrogels were placed in a refrigerator at a temperature of −26 °C for 24 h to later be introduced into a freeze dryer (SCIENTZ-10N LYOPHILIZER, Kansas City, MO, USA) for 24 h at 58 °C and pressure of 1 Pa [17]. Once the hydrogels were obtained, they were mechanically crushed for 5 min to obtain the microparticulate materials.

### 3.4. Physicochemical Characterization of Microparticles

#### 3.4.1. Scanning Electron Microscope (SEM) and Optical Microscopy (OM)

The surface morphology and size of the microparticles were analyzed by scanning electron microscope (SEM) and optical microscopy (OM). For SEM, Hitachi SU8230 equipment at 10 kV was used. The samples were mounted on aluminum tape and coated with gold, using Desk II high vacuum model LLC equipment. For OM, a LABOMED Model CX, Lamp 12 V 20 W device from Labo America, Inc., Fullerton, CA, USA was used. An OEM model objective micrometer was used to measure the apparent length of the microparticles.

#### 3.4.2. Specific Surface Area (SSA)

Surface specific area (SSA) is a property of solids defined as the total surface area of a material per unit of mass, this physical value is crucial in the characterization of pharmaceutical powders since it can provide information about their bioactive properties [56,57,58]. The analysis of SSA of the microparticles was carried out by nitrogen adsorption at 77 K to determine the specific surface area of the scaffolds, using Quentachome NOVA 2200e instruments Boynton Beach, FL, USA. The samples were previously degassed for 24 h, at 90 °C, to eliminate air and humidity present in the samples. The Brunauer–Emmett–Teller (BET) model was used to calculate the specific surface area of the materials obtained [59].

#### 3.4.3. Fourier Transform Infrared Spectroscopy (FTIR)

Fourier Transform Infrared Spectroscopy (FTIR) is used to monitor encapsulation processes since it allows analyzing the possible chemical bonds established between the encapsulating polymer and the active ingredient [60,61,62]. The spectra of analysis of the microparticles were obtained using a Perkin Elmer Spectrum Two FTIR spectrometer Fremont, CA, USA. A spectrum was analyzed in the wavenumber range of 4000–650 cm^−1^ with a resolution of 4 cm^−1^ and a ratio of 100 scans.

#### 3.4.4. Thermalgravimetric Analysis (TGA)

Thermogravimetric Analysis (TGA) helps to understand the thermal decomposition and the stability of formulations at high temperatures [63,64]. The thermogravimetric analysis of the microparticles was carried out in a Mettler Toledo TGA/DSC 2+ thermal analyzer (Greifensee, Switzerland), with a nitrogen atmosphere. Microparticles were measured between 25 and 600 °C with a heating ramp of 10°/min.

#### 3.4.5. Moisture Absorption

The analysis of the effect of humidity on formulation prototypes helps to understand their behavior in demanding environmental conditions. Humidity absorption is a gravimetric method that monitors the change in the humidity content of a polymer over time [65]. Its behavior also tells us if the material is hydrophilic or hydrophobic [66]. To perform the analysis, the freeze-drying process microparticles were stored under two relative humidity percentages (RH): 40 ± 1% and 80 ± 1%, using magnesium nitrate hexahydrate (Mg (NO_3_)_2_·6H_2_O) and sodium sulfate decahydrate (Na_2_SO_4_·10H_2_O), respectively. The mass of each of the samples was taken at the beginning of the study until equilibrium was reached. The percentage of moisture absorption (M. A.) is given by Equation (1):

(1) $$A\left(t\right)=\left(\frac{mt-m0}{m0}\right)\times 100$$

where *m*0 is the mass of the sample at the beginning of the exposure to moisture and *mt* is the mass of the sample after a time *t* in days. The mean and standard deviation are obtained in triplicate for each microparticle.

### 3.5. In Vitro Analysis of Microparticles

#### 3.5.1. Sample Sterilization

The samples were sterilized by exposure to UV radiation for 20 min using a laminar flow hood (Labconco^®^, Class II type A2, Kansas City, MO, USA).

#### 3.5.2. Cell Culture

Macrophages transformed by Abelson leukemia virus (RAW 264.7) were provided by Dr. Emil A. Unanue from the Department of Pathology and Immunology, Washington University, St. Louis, MO. USA. The RAW 264.7 cell line (Abelson leukemia virus-induced murine macrophages) was cultured in DMEM (Dulbecco’s modified Eagle’s medium) supplemented with 5% heat-inactivated fetal bovine serum and penicillin (100 U/mL) in 25 cm^2^ culture plates. Incubation was performed in an Isotherm incubator under the following conditions: 5% CO_2_, 37 °C and 95% relative humidity (Thermo Fisher Scientific, Waltham, MA, USA).

#### 3.5.3. Cytotoxic Effect

After RAW 264.7 cells reached 80% of confluence, they were detached by enzymatic digestion with 3 mL of trypsin and collected in DMEM. Subsequently, the cells were centrifuged (Beckman Coulter^®^ Allegra X-15R with SX4750 rotor, Brea, CA, USA) for 7 min at 1700 rpm and 4 °C. The supernatant was discarded, and the cell pellet was re-suspended with DMEM. A RAW 264.7 cell suspension with 500,000 cells/mL (cell viability *>* 90%) was placed in a 96-well plate (Costar, Corning, NY, USA) and incubated for 24 h. Subsequently, the cells were treated with the microparticles 0–200 μg/mL and 1 μg/mL of LPS (lipopolysaccharide) for 24 h by the indirect method (the microparticles were placed in a pH 5 buffer for 4 h and then 100 μL of the supernatant was taken to treat the cells). Then, 5 mg/mL of MTT (3-(4,5-dimethylthiazol-2-yl)-2,5-diphenyltetrazolium bromide) was added to lead to incubation for 4 h. Finally, the formazan crystals were resuspended with isopropanol and read at an absorbance at 570–630 nm in a model iMARK BIO-RAD^®^ (Gurgaon, India) microplate reader. Concentrations of the extracts tested where 90% or more cell viability was present are considered non-cytotoxic [67].

#### 3.5.4. Quantification of Nitric Oxide (NO) Production

The level of NO production will be analyzed in the supernatant of LPS-activated RAW 264.7 cells using Griess reagent. The cell suspension (50,000 cells/mL) was seeded in a 96-well plate (Costar, Corning, NY, USA) and incubated for 24 h. Subsequently, the cells are stimulated or not with LPS (1 μg/mL) in the presence or absence of the microparticles. Subsequently, 100 μL of the supernatant is mixed with an equal volume of Griess reagent and the reaction is subsequently incubated for 10 min at room temperature. Finally, the absorbance is read at 540 nm in a microplate reader (iMARK BIO-RAD^®^ microplate reader). A nitrite standard curve is used to determine NO levels. IC_50_ values (concentration required to inhibit 50% of NO production) are calculated using linear regression [67].

#### 3.5.5. Antimicrobial Activity

Antimicrobial evaluation of microparticles loaded with *B. microphylla* A. Gray fruit extract was performed using the method described by CLSI. Overnight culture bacteria (37 °C) was adjusted at 0.5 McFarland (1 × 10^8^ CFU/mL). Afterward, 0.1 mL of the bacterial suspension was mixed with 9.9 mL of Mueller Hinton broth reaching a concentration of 1 × 10^6^ CFU/mL. Subsequently, microparticles loaded with *B. microphylla* extract were mixed with Mueller Hinton broth. A 1:1 proportion of bacterial inoculum and microparticles was combined and incubated for 24 h at 37 °C. Microparticles without *B. microphylla* extract was used as a control. After, 50 µL of each treatment was combined with saline solution (0.85% *w/v*) in a proportion 1:10, performing serial dilution. Each dilution (10 µL) was plated on plate count agar. The plates were incubated for 24 h at 37 °C. Finally, the results were reported as CFU/mL after the colony’s enumeration.

### 3.6. Statistical Analysis

Data were expressed as mean ± standard deviation. Statistical analysis was performed via a one-way analysis of variance (ANOVA) followed by a Tukey’s test in the GraphPad PRISM^®^ version 6.0c program, Boston, MA, USA. A value of *p* < 0.05 was considered statistically significant.

## 4. Conclusions

In this research, it was possible to synthesize Cs microparticles loaded with *B. microphylla* fruit extract with anti-inflammatory and antimicrobial effects. The clinical implications of these findings suggest that chitosan-based microparticles could serve as a safer alternative to traditional NSAIDs, reducing the risk of gastrointestinal and cardiovascular side effects associated with prolonged use and could be an alternative for the treatment of infections caused by *S. aureus*.

More studies are needed to assess the anti-inflammatory effect in vivo models to continue profiling the CsB-0.5 end CsB-1.0 encapsulating system of *B. microphylla*, as a pharmaceutical candidate for the treatment and control of inflammation and infections caused by *S. aureus*.

### Future Prospects

Future studies will focus on preclinical evaluations in animal models to further assess the efficacy and safety profile of these microparticles, paving the way for clinical trials.

The scalability of the production process for these chitosan-based microparticles is another critical consideration. Future work will focus on optimizing the synthesis process to ensure cost-effectiveness and reproducibility at an industrial scale.

While chitosan is a well-known biopolymer, exploring the combination with other natural polymers such as alginate or gelatin could further enhance the stability and bioactivity of the encapsulated compounds.

## Figures and Tables

**Table 1:** Specific surface area (SSA) of encapsulating materials with chitosan as polymer matrix.

Composition	SSA (m^2^/g)	Reference
Cs	05.753	This work
CsB-0.5	12.111	This work
CsB-1.0	03.220	This work
Cs-ethylene glycol diglycidyl ether	00.620	[28]
Cs-sodium lauryl sulphate	00.670	[29]
Cs	01.200	[30]
Carboxymethyl-Cs	00.490	[31]
Tin (IV)-Cs	0.3020	[32]
Polyvinyl alcohol-Cs	01.948	[33]
Polyethylene glycol-Cs	02.679	[33]
Aliquant 336-Cs	02.430	[34]

Cs: chitosan; B: *B. microphylla* extract.

## Data Availability

The raw data supporting the conclusions of this article will be made available by the authors on request.
